# A Cup of Hemp Coffee by Moka Pot from Southern Italy: An UHPLC-HRMS Investigation

**DOI:** 10.3390/foods9081123

**Published:** 2020-08-14

**Authors:** Simona Piccolella, Giuseppina Crescente, Marialuisa Formato, Severina Pacifico

**Affiliations:** Department of Environmental, Biological and Pharmaceutical Sciences and Technologies, University of Campania “Luigi Vanvitelli”, Via Vivaldi 43, 81100 Caserta, Italy; simona.piccolella@unicampania.it (S.P.); giuseppina.crescente@unicampania.it (G.C.); marialuisa.formato@unicampania.it (M.F.)

**Keywords:** hemp inflorescences, coffee, moka, UHPLC-HRMS, phytocannabinoids, chlorogenic acids

## Abstract

After a long period defined by prohibition of hemp production, this crop has been recently re-evaluated in various industrial sectors. Until now, inflorescences have been considered a processing by-product, not useful for the food industry, and their disposal also represents an economic problem for farmers. The objects of the present work are coffee blends enriched with shredded inflorescences of different cultivars of industrial hemp that underwent solid/liquid extraction into the Italian “moka” coffee maker. The obtained coffee drinks were analyzed by Ultra-High-Performance Liquid Chromatography-High Resolution Mass Spectrometry (UHPLC-HRMS) tools for their quali-quantitative phytocannabinoid profiles. The results showed that they are minor constituents compared to chlorogenic acids and caffeine in all samples. In particular, cannabidiolic acid was the most abundant among phytocannabinoids, followed by tetrahydrocannabinolic acid. Neither Δ^9^-tetrahydrocannabinol (THC) nor cannabinol, its main oxidation product, were detected. The percentage of total THC never exceeded 0.04%, corresponding to 0.4 mg/kg, far below the current maximum limits imposed by the Italian Ministry of Health. This study opens up a new concrete possibility to exploit hemp processing by-products in order to obtain drinks with high added value and paves the way for further in vitro and in vivo investigations aimed at promoting their benefits for human health.

## 1. Introduction

The long coexistence between mankind and *Cannabis sativa* L. led to its early domestication, with the plant soon showing a plethora of possible uses, making it an emblematic example of polyvalent culture [1]. Over the centuries, this plant has gone from robust and durable textile fibers to recreational drugs used by artists and writers in the 19th century [2], until the beginning of a long period defined by the prohibition of its production, which continued until recent years. In fact, the contemporary re-evaluation of this crop has been stimulated by a number of studies that highlighted its agricultural features, together with its beneficial properties due to its undervalued richness in phytochemicals, in addition to fiber. In this context, many countries have authorized the cultivation and processing of hemp varieties (generally referred to as industrial hemp) with very low content of psychotropic compounds [3].

In particular, the food chain has mainly considered hemp seed intake, as well as that of the oil extracted therefrom and baked products derived from flour. Several studies crowned the hemp seed as a rich source of nutrients with health-promoting properties. The chemical composition and the nutraceutical nature of these fruits have been recently reviewed, pointing out that its functional benefits are still far from being fully understood [4]. In fact, besides essential polyunsaturated fatty acids with a ratio nearly equal to 3:1 (ω-6: ω-3), which characterize also the oil together with vitamins, minerals, amino acids and phytosterols, the presence of antioxidant (poly)phenols is worth mentioning, not only for their preservation of oil stability and quality but also for their numerous health benefits for consumers [5,6,7].

As the commercial interest (from farmers to producers) has increased, at the same time, the safety of products intended for human consumption has begun to be regulated at the legislative level. Italian legislation, with the circular of the Ministry of Health on 23 May 2009, has allowed the production and marketing of hemp seed products for use in the human nutrition sector, thus allowing the use of *C. sativa*, or parts thereof, in foodstuffs such as bread flour, sweets, oils and supplements [8]. Then, other decree laws followed, all focused on the *Cannabis sativa* plant and its cultivation. The first Italian regulation which fixed the maximum permitted levels of total THC in hemp-derived food dates back to 4 November 2019 and refers to hemp seeds (2.0 mg/kg), the oil (5.0 mg/kg) and flour therefrom (2.0 mg/kg) and also hemp-based supplements (2.0 mg/kg) [9].

Indeed, the growing expansion of marketed hemp foodstuffs, beyond seeds and derived products, requires the development and application of multiple quantitative analytical methods to determine the cannabinoid content in a wide range of matrices. Gas chromatography (GC) coupled with mass spectrometry (MS) or a flame ionization detector (FID), although widely used, suffers limitations in its ability to identify and quantify acid cannabinoids (e.g., cannabigerolic acid—CBGA, cannabidiolic acid—CBDA, and tetrahydrocannabinolic acid—THCA), because, during the analyses, they could decarboxylate in their neutral forms, due to high temperatures [10]. In this sense, liquid chromatography (LC) appears to be a more suitable tool for analyzing the native composition of the hemp plant. For the quantification of both acid and neutral cannabinoids, methods based on HPLC-MS, HPLC with a Diode-Array Detector (DAD) and HPLC-DAD-MS techniques have been reported, as well as the application of supercritical fluids in chromatographic separation (SFC-DAD-MS), developed for plant materials and plant extracts/oils, including those derived from illicit, medicinal and/or industrial varieties [11]. In particular, LC separation combined with tandem mass spectrometry (LC-MS/MS) with electrospray ionization (ESI) could be considered as the method of choice in the quali-quantitative determination of phytocannabinoids present in very low amounts, mainly due to the high signal-to-noise ratio and selectivity. For accurate mass estimation, time of flight (TOF) or linear ion trap-Orbitrap (LTQ-Orbitrap) hybrid mass analyzers have been successfully used [12].

In light of the above, herein, we applied ultra-high-performance liquid chromatography (UHPLC) techniques with high resolution tandem mass spectrometry (HR-MS/MS) detection to the analysis of three espresso coffees from mixtures of ground coffee enriched with shredded inflorescences from three different dioecious genotypes of *C. sativa* L. (*cvs*. Antal, Kompolti and Tiborszallasi), commonly cultivated for industrial purposes. The thermodynamics measurement of the coffee extraction process by “moka” (the most common Italian coffee maker) led to the distinction of two distinct phases [13]. In the first one, called “regular extraction”, liquid–solid extraction occurs, which is characterized by variable temperature and water flow rate over time. In this phase, the extraction is guided by the increase of the air vapor pressure above the water level in the lower tank of the device. In the second phase, known as the “strombolian phase”, intense evaporation takes place, resulting in the extraction of soluble compounds which are generally detrimental to the quality of the final product.

The experimental work described herein was aimed at the chemical characterization of the main phytocannabinoids extracted during the “moka” process and their quantification in the coffee drink obtained, also compared to the natural phenolic constituents of coffee (chlorogenic acids) and caffeine, concluding with discussion of the possible benefits deriving from this enrichment. In addition, the targeted analysis of neutral phytocannabinoids allowed us to verify possible decarboxylation of the acid cannabinoids during the “extraction” process due to high temperature and pressure. This study opens up a new concrete possibility to raise industrial hemp inflorescences, converting the by-product production chain into a high value chain.

## 2. Materials and Methods

### 2.1. Coffee Extraction by the “Moka” Stove-Top Coffee Maker

Three different coffee blends enriched with shredded inflorescences from different cultivars of industrial hemp (*cvs*. Antal, Kompolti and Tiborszallasi), namely Canffé, distributed at the local market and available for sale online, were considered. Three coffee drinks were obtained therefrom by means of the Italian stove-top coffee maker known as “moka”. Briefly, for solid–liquid extraction purposes, 15 g of each ground coffee variety were placed in a 3-cup coffee pot, whose boiler was filled up to the safety valve (150 mL of water). Extractions were conducted in triplicate. Immediately after the injection in the ultra-high-pressure liquid chromatography electrospray ionization quadrupole time-of-flight mass spectrometry (UHPLC-ESI-QqTOF MS) system, the coffee drinks were freeze-dried using the FTS System Flex-DryTM instrument (SP Scientific, Stone Ridge, NY, USA), in order to express metabolite amounts as percentages referring to their dry weight.

### 2.2. UHPLC-ESI-HRMS Parameters

The coffee drinks obtained were directly injected in the the Shimadzu NEXERA^®^ UHPLC system using the Omega Luna^®^ C18 column (50 × 2.1 mm i.d., 1.6 μm particle size). The mobile phase consisted of a binary solution composed by water (solvent A) and acetonitrile (solvent B), both acidified with formic acid (0.1% *v*/*v*). A linear gradient was used, which was as follows: 0–5 min, 5→15% B; 5–10 min, 15% B; 10–12 min, 15→17.5% B; 12–15 min, 17.5→45% B; 15–16 min, 45→55% B; 16–21 min, 55→75% B; 21–22 min, 75→95% B; 22–23 min, 95% B. Then, the system was allowed to re-equilibrate before the next analysis. The injection volume was 2.0 μL and the flow was set at 0.4 mL/min.

The AB SCIEX TripleTOF^®^ 4600 (AB Sciex, Concord, ON, Canada) system was combined with the UHPLC. It was equipped with a DuoSpray ion source, with the ESI probe used for MS investigations in both negative and positive ionization mode, and the APCI probe used for fully automatic mass calibration, using the Calibrant Delivery System (CDS). CDS injects a calibration solution matching the polarity of ionization and calibrates the mass axis of the analyzer in all scan functions (MS or MS/MS). Data were collected by information dependent acquisition (IDA) using a TOF-MS survey scan of 100–1500 Da (250 ms accumulation time) and eight dependent TOF-MS/MS scans of 80–1200 Da (100 ms accumulation time), using a collision energy (CE) of 45 V with a collision energy spread (CES) of 15 V. The other parameters were set as follows: declustering potential (DP), 70 V; ion spray voltage, −4500 (+5500) V; ion spray heater, 600 °C; curtain gas, 35 psi; ion source gas, 60 psi. Data processing was performed using the PeakView^®^-Analyst^®^ TF 1.7 software.

### 2.3. Quantification of Phytocannabinoids, Chlorogenic Acids and Caffeine

Phytocannabinoids, chlorogenic acids and caffeine were quantified in coffee drinks by means of the external standard method. The reference standards caffeine, cannabidiol (CBD) and 5-*O*-caffeoylquinic acid (5-CQA) were purchased from Sigma-Aldrich (Milan, Italy), whereas CBDA and THCA were previously isolated in our lab [14]. For this purpose, calibration curves were constructed (Table 1), injecting working solutions of each standard, prepared by dilution from a stock solution, into the UHPLC-ESI-QqTOF MS system under the same conditions as the samples.

The analyses were conducted in three independent measurements and the results, expressed as wt % of dried coffee drinks, represented mean values ± standard deviation (SD). The total THC and CBD content were calculated, according to the Italian legislation, with the following equations:

Total THC % = (THCA %) × 0.877 + (THC %)(1)

Total CBD % = (CBDA %) × 0.877 + (CBD %)(2)

## 3. Results and Discussion

Coffee, with its intense aroma and creamy texture, is one of the most popular drinks in the world and enriches the traditions of countless countries. In Italy, the most popular domestic method of preparation uses a kitchen coffee maker, in which steam pressure, produced in an aluminum kettle containing water warmed up by an external source, is forced through a bed of roasted and ground coffee, contained in a funnel-shaped filter [13].

The work herein reported is focused on three moka coffee drinks obtained from ground coffee blends, enriched with shredded inflorescences from three different dioecious genotypes of *C. sativa* L. (*cvs*. Antal, Kompolti and Tiborszallasi), marketed in Southern Italy and available for sale online (e.g., ebay website). The three coffee drinks underwent ultra-high-performance liquid chromatography analysis, combined with high resolution mass spectrometry (UHPLC-HRMS). The aim was the chemical characterization of the phytocannabinoids extracted during the moka process and their quantification, also in relation to the amounts of the naturally occurring coffee metabolites, i.e., chlorogenic acids and caffeine.

It is worth remembering that the hemp plant biosynthesizes the cannabinoids in the acidic form. They are thermally unstable and can be decarboxylated if exposed to light or heating. Recently, the decarboxylation of acidic cannabinoids at different temperatures (between 80 and 145 °C) for different times (up to 60 min) was studied by ultra-high performance supercritical fluid chromatography/photodiode array-mass spectrometry (UHPSFC/PDA-MS) [15], demonstrating an exponential relationship between concentration and time, which indicates a first or pseudo-first order reaction, which could be catalyzed by some acids naturally occurring in the plant [16].

The fragmentation pattern of each detected metabolite was investigated, not only in terms of *m*/*z* ratio but also, and above all, taking into careful consideration the fragments’ intensity, obtained during the collision-induced dissociation (CID) of the precursor ions. This is particularly important in the case of constitutional isomers, which often give rise to qualitatively similar MS/MS spectra. Therefore, the only possibility of discrimination is the assessment of relative ionic abundance, calculated by taking into account the base peak of the spectrum. Indeed, a deep rationalization of fragmentation pathways is essential to delineate molecular structures and the connectivity of functional groups, up to the compounds’ “identity card” [17]. The use of a hybrid QqTOF mass analyzer proved to be very effective in this regard, as it can provide high resolution MS/MS spectra with good mass accuracy (errors below 5 ppm), both for the precursor ion and for the fragments.

### 3.1. Quali-Quantitative Analysis of the Main Phytocannabinoids

**Figure 1:**
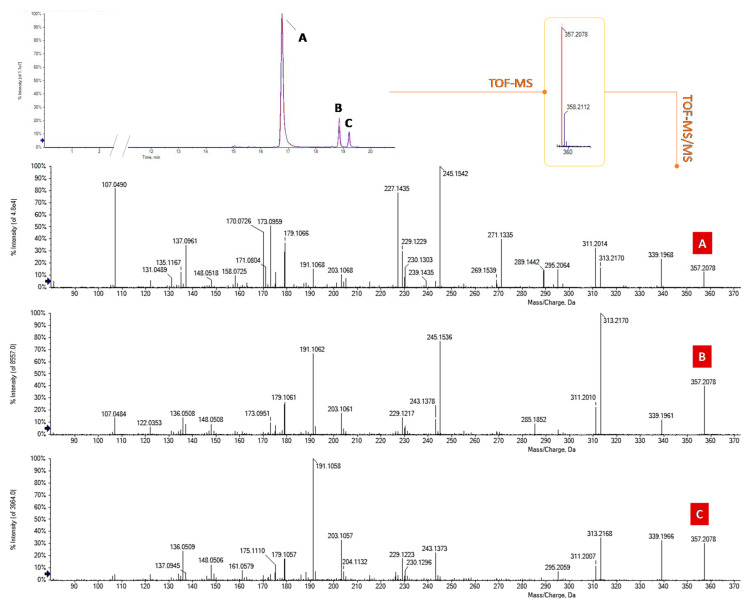
Overlapped XICs (extracted ion chromatograms) of the acidic phytocannabinoids at *m*/*z* 357.20 ± 0.025 in the three coffee drinks investigated and their TOF-MS^2^ spectra. (**A**) Cannabidiolic acid (CBDA); (**B**) Δ^9^-Tetrahydrocannabinolic acid (THCA-A); (**C**) Cannabichromenic acid (CBCA).

The targeted UHPLC-MS analysis in negative ion mode initially focused on the search for precursor ions at *m*/*z* 357.20 belonging to the most famous acidic phytocannabinoids, CBDA and THCA, whose presence is ubiquitous in the different cultivars, even if in different amounts, depending on the plant chemotype and the pedoclimatic growth conditions. Figure 1 shows the overlapped XICs (extracted ion chromatograms) of the three coffee drinks investigated, obtained by selecting this ion ±0.025 Da. The chromatograms show three well-resolved peaks. The *m*/*z* value of the deprotonated precursor ions was in accordance with the molecular formula C_22_H_30_O_4_ and the RDB (ring double bonds) value was equal to 8. TOF-MS^2^ spectra differed mainly in the relative ionic intensities (Figure 1A–C).

Based on comparison with a pure reference compound, previously isolated and chemically characterized by means of spectroscopic and spectrometric techniques [14], the compound eluting at the lower retention time (A; 16.8 min) was identified as cannabidiolic acid (CBDA-C5). This latter is the acidic phytocannabinoid mainly produced by industrial *Cannabis sativa* L. plant varieties. TOF-MS^2^ data promptly favored the compound recognition. In fact, the deprotonated molecular ion undergoes dehydration or, alternatively, decarboxylation, generating the ions at *m*/*z* 339.1966 and 313.2173, respectively. When decarboxylation is followed by the loss of an isoprene unit (68 Da), the ion at *m*/*z* 245.1542 is generated. It represents the base peak of the MS/MS spectrum, unlike the other two isomers (Figure 1). Moreover, the ions at *m*/*z* 227.1435 and 271.1335 could be considered diagnostic in the discrimination between CBDA and its constitutional isomers, in whose MS/MS spectra they are absent and/or lower than 2%. Compound B (Figure 1) was identified as Δ^9^-THCA, also called THCA-A or simply THCA. In this case, the fragmentation reaction that generated the base peak (at *m/z* 313.2170) is decarboxylation, and the intensity ratio between this ion and the one at *m*/*z* 339.1961 ([M-H-H_2_O]^−^), which is >> 1, confirmed the hypothesis of a Δ^9^-THC type structure [18].

Finally, the fragmentation pattern of isomer C (Figure 1), eluting at 19.2 min, was in agreement with the presence of a CBC-type cannabinoid, for which the base peak was the ion at *m*/*z* 191.1058, resulting from the neutral loss of 166.102 Da ([M-H-CO_2_-C_5_H_8_-C_4_H_6_]^−^). Thus, it has been tentatively identified as CBCA.

**Figure 2:**
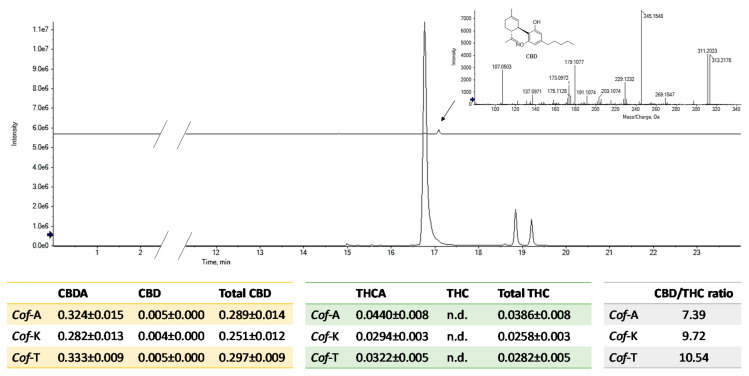
XICs of acidic cannabinoids CBDA, THCA-A and CBCA and of neutral CBD. In the tables, the amount of each compound is reported as wt % of dried moka coffee drinks (n.d. = not detected; *Cof*-A = coffee enriched with *C. sativa* cv. Antal shredded inflorescences; *Cof*-K = coffee enriched with *C. sativa* cv. Kompolti shredded inflorescences; *Cof*-T = coffee enriched with *C. sativa* cv. Tiborszallasi shredded inflorescences).

As ionization efficiency is closely related to the chemical nature of a compound, quantitation was carried out by using, as external standards, pure commercial cannabidiol (CBD) and CBDA and THCA, previously isolated in our lab (CBDA). Quantitative analysis showed that, in the three investigated coffee drinks, the most abundant acidic phytocannabinoid is CBDA, followed by THCA and finally by CBCA (Figure 2). Among neutral cannabinoids, CBD is the only one identified, whereas no trace of Δ^9^-THC could be detected.

Qualitative analysis underlines that the three constitutional isomers CBDA, THCA and CBCA were the most representative compounds. Quantitation data underline that the total CBD/THC ratio approximately ranged from 7:1 to 10:1. The minimal presence of cannabidiol, the only neutral phytocannabinoid found in the studied coffee drinks, does not seem to be due to a decarboxylation process during moka extraction, considering the maximum temperatures reached and the very limited period of exposure. Since the plant produces only acid cannabinoids [19], it is likely that CBDA→CBD transformation occurred during the post-harvest storage period of the plant material before use. However, no trace of Δ^9^-THC or cannabinol (CBN), the main oxidation product of Δ^9^-THC in the presence of oxygen and light, was detected. Moreover, the total THC content was calculated, as required by the current Italian legislation, and also the total CBD. In all the investigated coffee drinks, the total THC never exceeded 0.04%, corresponding to 0.4 mg/kg. This value is far below the maximum limits currently imposed by the Italian Ministry of Health (2.0 mg/kg in hemp-based supplements [9]); therefore, it ensures that the products meet legal requirements. Indeed, the CBD/THC ratio could depend on different factors, e.g., genetic characteristics of *C. sativa* chemotype, growth conditions and age of the plant at harvest time [20]. In recent literature, this variable ratio was taken into account for different pharmacological purposes. In fact, high CBD/THC (up to 18:1) products are mainly useful for anxiety, depression, psychosis and other mood disorders; instead, low CBD/THC (1:1) products are used to relieve neuropathic pain and rheumatism [21]. The anxiolytic effects of CBD (and CBDA) appear to be mediated by 5-HT1A receptor activation [22,23], unlike Δ^9^-THC, which modulates anxiety by CB1 receptor interaction [24]. Although scientific literature in the past has focused more on the pharmacological effects of CBD [25], highlighting its multiple biological activities and considering CBDA only as a biosynthetic precursor in the plant, in the last decade, some experimental evidence suggests that CBDA is the main actor [14]. One of the most studied effects concerns its ability to reduce emetic response, including that due to chemotherapy [26,27,28,29]. Some studies have shown that CBDA is a potential migration inhibitor of highly invasive cancer cells, in particular the MDA-MB-231 cell line, which is responsible for the occurrence of breast cancer. This effect probably relies on the ability to inhibit a cAMP-dependent protein kinase with simultaneous activation of the small GTPasi RhoA [30]. Suzuki et al. [31] reported that CBDA is able to down-regulate the expression of cyclooxygenase isoform 2 (COX-2) in these cells. Besides inflammation, it is involved in metastasis processes, so much so that COX-2 expression has been detected in around 40% of invasive breast cancers; therefore, it was assumed that chemical inhibition and down-regulation of this enzyme could be a key factor in the inhibition of cell migration due to CBDA [32,33].

### 3.2. Minor Phytocannabinoids Constituents

**Figure 3:**
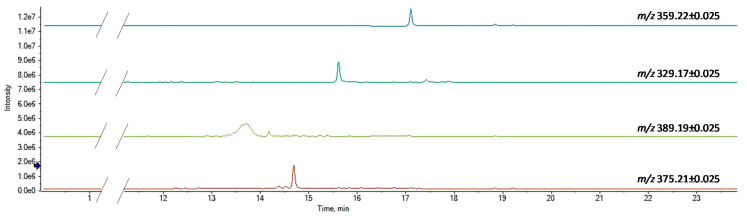
XICs of minor phytocannabinoid constituents.

The other phytocannabinoids detected in the three coffee drinks can be considered as minor components (Figure 3).

**Figure 4:**
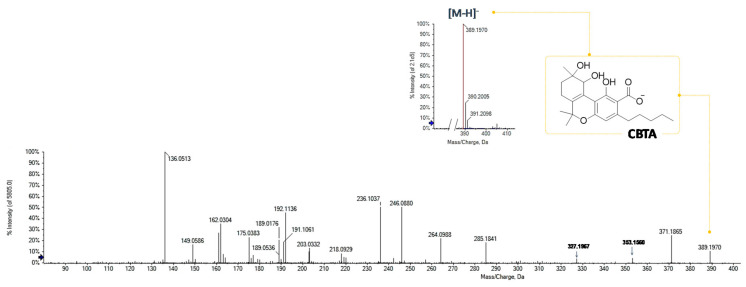
HR-MS and MS/MS of the phytocannabinoid at *m*/*z* 389.1970.

The MS/MS spectrum of the deprotonated compound at *m*/*z* 389.1970 (C_22_H_32_O_6_) appeared to be in accordance with an acidic CBT-type phytocannabinoid (Figure 4). In fact, the first fragmentations derive from the loss of two water molecules, to generate ions at *m*/*z* 371.1865 and 353.1760 [18]. Contrary to what was described for CBDA and its isomers, in this case, decarboxylation from the parental ion occurred only after dehydration (*m*/*z* 389.1970 → 371.1865 → 327.1967). This could be indicative of the formation of a strong hydrogen bond between the carboxylic group and the hydroxyl substituent at the vicinal carbon. Its retention time in reversed phase chromatography (13.7 min), lower than that of CBDA, is in agreement with greater polarity due to the presence of an additional OH on the monoterpenic moiety.

**Figure 5:**
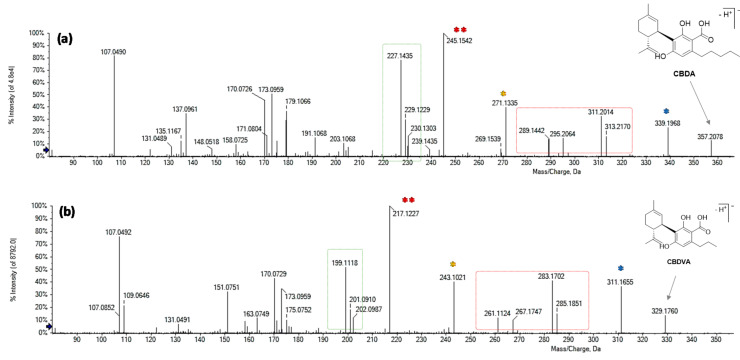
TOF-MS^2^ spectra of (**a**) CBDA and (**b**) putative CBDVA. Fragment ions deriving from the same neutral losses are marked with the same symbol.

The phytocannabinoid characterized by a [M-H]^−^ ion at *m*/*z* 329.1760 (Figure 3), whose proposed molecular formula is C_20_H_26_O_4_, was likely cannabidivarinic acid (CBDVA). In fact, comparing its TOF-MS^2^ spectrum with that of CBDA, similar neutral losses and relative intensities of the daughter ions were observed (Figure 5). Thus, the observed mass difference is likely due to the side alkyl chain, which, for CBDVA, is a propyl rather than a pentyl moiety.

**Figure 6:**
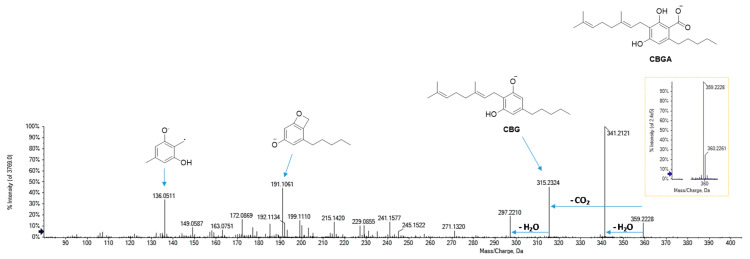
HR-MS and MS/MS spectra of CBGA (at *m*/*z* 359.2228).

Cannabigerolic acid (CBGA) was supposed to be the compound with the deprotonated parent ion at *m*/*z* 359.2228 (in accordance with the molecular formula C_22_H_32_O_4_) [34]. The base peak of its MS/MS spectrum (Figure 6) at *m*/*z* 341.2121 arose from dehydration, whereas a decarboxylation reaction led to the formation of the fragment ion at *m*/*z* 315.2324. From this latter, by cleavage of the monoterpenic chain, the product ion at *m*/*z* 191.1061 was formed, which gave rise to the radical ion at *m*/*z* 136.0535 by further α-scission of the pentyl side chain.

**Figure 7:**
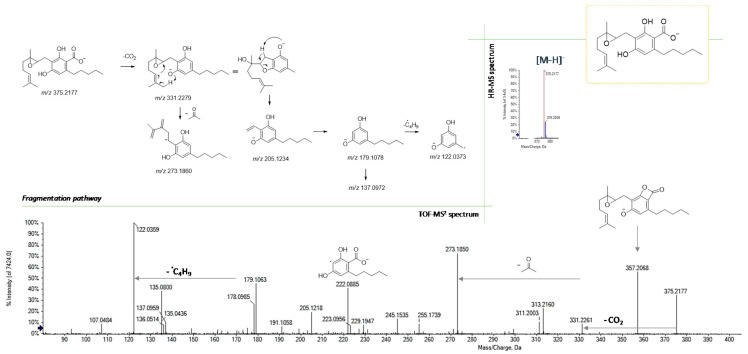
HR-MS and MS/MS spectra of 6,7-epoxyCBGA (at *m*/*z* 375.2177) and the proposed fragmentation pathway.

Finally, according to the molecular formula C_22_H_32_O_5_, the compound with the [M-H]^−^ ion at *m*/*z* 375.2177 showed one more oxygen atom than CBGA and was tentatively identified as its 6,7-epoxy derivative. In Figure 7, its TOF-MS^2^ spectrum and the hypothesized fragmentation pathway are presented. Besides the occurrence of dehydration and decarboxylation fragments, which are common to CBGA, the deprotonated olivetol unit at *m*/*z* 179.1063 is shown, which in turn generated the radical ion at *m*/*z* 122.0359 following the homolytic cleavage of the pentyl chain. Alternatively, the olivetolic acid radical anion at *m*/*z* 222.0885 could be obtained directly from the parent ion, due to the loss of the terpenoid moiety (−153.12 Da).

### 3.3. Caffeine and Chlorogenic Acids Content

The purine alkaloid caffeine and chlorogenic acids are considered the main bioactive compounds of coffee, along with cafestol and kahweol pentacyclic diterpenes, trigonelline and melanoidins [35,36]. Thus, besides phytocannabinoids, their content in investigated hemp coffee was also investigated.

Targeted UHPLC-ESI-HRMS in positive ion mode was a useful tool to detect and quantify caffeine in the three coffee drinks obtained by moka. The results showed a content level of this purine alkaloid equal to 2.225 ± 0.033 wt %, 1.970 ± 0.015 wt % and 3.021 ± 0.021 wt % with respect to dried samples (coffee enriched with *C. sativa* cvs. Antal, Kompolti and Tiborszallasi shredded inflorescences, respectively) (Figure S1).

Typical caffeine levels in a coffee cup vary on average from 50 to 100 mg, although some studies report amounts over 300 mg [37]. This variability depends on the preparation method and also on the coffee species; in fact, it has been reported that *Coffea canephora* (robusta) contains greater quantities than the *Coffea arabica* pecies [38]. Indeed, although the composition of the studied ground coffee mixture was not known, the values obtained, in line with the literature, seem to suggest a higher percentage of *var*. robusta. In fact, unlike other bioactive molecules of the *Coffea* fruits, the caffeine content does not vary particularly during the roasting process, and it has been estimated to be equal to 0.9–1.3% and 1.5–2.5% of the dry weight of coffee of Arabica and Robusta varieties, respectively [37]. Once absorbed, caffeine shows numerous physiological effects [39], mainly through antagonism of the adenosine receptors A1 and A2. The antagonism towards adenosine increases dopamine levels, which are responsible for many of the stimulating properties in the central nervous system and dependence on caffeine. Another mechanism of action of caffeine is based on synergistic interaction with adrenaline and noradrenaline, the main neurotransmitters for the sympathetic nervous system [40]. The stimulating effects of caffeine include better perception, greater ability to stay awake for longer periods and reduced fatigue. More recently, caffeine has been shown to have the positive effect of improving memory consolidation [41]; it also helps to reduce symptoms associated with Parkinson’s disease [42] and was suggested to be involved in mitigating the risk factors of metabolic syndrome and obesity [39]. An interesting correlation between the antioxidant capacity of coffee-based drinks and caffeine was found [43], suggesting its potential contribution in reducing pro-oxidant agents in the human body.

However, traditionally, the high antioxidant capacity of coffee has been attributed to its phenolic component and, in particular, to the presence of chlorogenic acids, namely esters of quinic acid with cinnamic acids (such as caffeic, ferulic or *p*-coumaric acid). They proved to have several positive effects on human health: they are able to delay the absorption of glucose in the intestine, to improve the antioxidant balance of the body, to reduce low-density lipoproteins (LDL) oxidation and to slow down the inflammatory process [44]. Targeted UHPLC-ESI-HRMS in negative ion mode allowed us to recognize and quantify the main chlorogenic acids found in the coffee-based drinks under study. The structures of these compounds, which are also found in the fruits of the coffee plant, are depicted in Figure S2.

From a chemical point of view, they can be divided into different subclasses according to the nature, number and positions of the cinnamic substituents. In particular, five mono-caffeoylquinic acids (CQAs), six dehydrated derivatives, mainly caffeoylshikimic acids and/or caffeoylquinic acid lactones (CSAs; CQLs), four di-caffeoylquinic acids (di-CQAs) and five feruloylquinic acids (FQAs) were putatively identified (Figures S3–S6).

**Figure 8:**
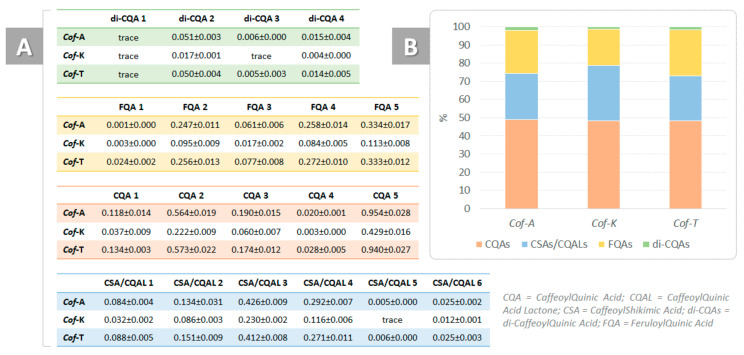
(**A**) Chlorogenic acids amounts, expressed as wt % of dried moka coffee drinks (*Cof*-A = coffee enriched with *C. sativa cv*. Antal shredded inflorescences; *Cof*-K = coffee enriched with *C. sativa cv*. Kompolti shredded inflorescences; *Cof*-T = coffee enriched with *C. sativa cv*. Tiborszallasi shredded inflorescences); (**B**) % of the four subclasses in each sample.

Although it has been reported that most of them are degraded during the roasting process [45], they are still the main phenolic components of coffee. In all the samples under study, CQAs constituted the most abundant subclass (Figure 8), representing around 50%, whereas di-CQAs were the least abundant. Furthermore, dehydrated derivatives—that is, CSAs and/or CQLs—accounted for 25–30% of the total chlorogenic acids and were likely formed during the coffee roasting process [46]. The chemical characterization and the distinction between all the possible regioisomers do not fall within the scope of the present work, as several papers in the literature have already been dedicated to this topic [47,48,49,50,51,52,53,54,55].

## 4. Conclusions

The actual renewed interest in hemp cultivation is related to the so-called industrial hemp varieties, which, characterized by very THC low content, are used for the production of fibers or seeds and derived food products such as seed oil or flour. In this context, the inflorescences of the plant represent a by-product of the production chain. The enrichment of coffee blends with shredded inflorescences from industrial hemp—Until now, not considered useful for the food industry—Opens up a new concrete possibility to exploit hemp processing by-products to obtain drinks with high added value, where the stimulating effects of caffeine and the high antioxidant capacity attributed to the phenolic component could combine the positive effects (combating anxiety, depression, psychosis and other mood disorders) potentially attributable to the CBDA/THCA ratio. Therefore, this study paves the way for further in vitro and in vivo investigations aimed at promoting hemp coffee benefits for human health.

## Figures and Tables

**Table 1:** Calibration Curves Used for Quantitation Purposes.

Compound	Linearity Range	Equations	R^2^
CBD	0.835–33.4 ng *	*y* = 2 × 10^8^ *x*	0.9942
CBDA	3.9–31.2 ng *	*y* = 2 × 10^8^ *x* + 1 × 10^6^	0.9918
THCA	0.12–15.6 ng *	*y* = 3 × 10^8^ *x* + 99273	0.9992
5-CQA	6.25–250 ng *	*y* = 5 × 10^7^ *x* + 180118	0.9959
Caffeine	0.56–2.26 μg *	*y* = 1 × 10^10^ *x* + 2 × 10^7^	0.9995

* on column amounts. CBD: cannabidiol CBDA: cannabidiolic acid THCA: tetrahydrocannabinolic acid 5-CQA: 5-O-caffeoylquinic acid.

## Supplementary Materials

The following are available online at https://www.mdpi.com/2304-8158/9/8/1123/s1, Figure S1: XIC, HR-MS and MS/MS of caffeine. In the table, its amount is reported as wt % of dried moka coffee drinks (Cof-A = coffee enriched with *C. sativa cv*. Antal shredded inflorescences; Cof-K = coffee enriched with *C. sativa cv*. Kompolti shredded inflorescences; Cof-T = coffee enriched with *C. sativa cv*. Tiborszallasi shredded inflorescences), Figure S2: Chemical structures of the main chlorogenic acids (CQA = caffeoylquinic acid; CQAL = caffeoylquinic acid lactone; FQA = feruloylquinic acid; FQAL = feruloylquinic acid lactone), Figure S3: XIC (selecting the ion at *m*/*z* 353.09 ± 0.025) and HR-MS/MS of monocaffeoylquinic acids (CQAs) (Cof-A = coffee enriched with *C. sativa cv*. Antal shredded inflorescences; Cof-K = coffee enriched with *C. sativa cv*. Kompolti shredded inflorescences; Cof-T = coffee enriched with *C. sativa cv*. Tiborszallasi shredded inflorescences), Figure S4: XIC (selecting the ion at *m*/*z* 515.10 ± 0.025) and HR-MS/MS of di-caffeoylquinic acids (di-CQAs) (Cof-A = coffee enriched with *C. sativa cv*. Antal shredded inflorescences; Cof-K = coffee enriched with *C. sativa cv*. Kompolti shredded inflorescences; Cof-T = coffee enriched with *C. sativa cv*. Tiborszallasi shredded inflorescences), Figure S5: XIC (selecting the ion at *m*/*z* 335.08 ± 0.025) and HR-MS/MS of dehydrated derivatives of caffeoylquinic acids (CSAs/CQALs) (Cof-A = coffee enriched with *C. sativa cv*. Antal shredded inflorescences; Cof-K = coffee enriched with *C. sativa cv*. Kompolti shredded inflorescences; Cof-T = coffee enriched with *C. sativa cv*. Tiborszallasi shredded inflorescences), Figure S6: XIC (selecting the ion at *m*/*z* 367.08 ± 0.025) and HR-MS/MS of feruloylquinic acids (FQAs) (Cof-A = coffee enriched with *C. sativa* cv. Antal shredded inflorescences; Cof-K = coffee enriched with *C. sativa cv*. Kompolti shredded inflorescences; Cof-T = coffee enriched with *C. sativa cv*. Tiborszallasi shredded inflorescences).
