# Supplementary material for: A Cup of Hemp Coffee by Moka Pot from Southern Italy: An UHPLC-HRMS Investigation
Source: Foods. 2020 Aug 14;9(8):1123. doi: 10.3390/foods9081123 (PMC7466224; doi:10.3390/foods9081123)
Supplement: Supplementary file 1 [file foods-09-01123-s001.pdf]

Supplementary materials

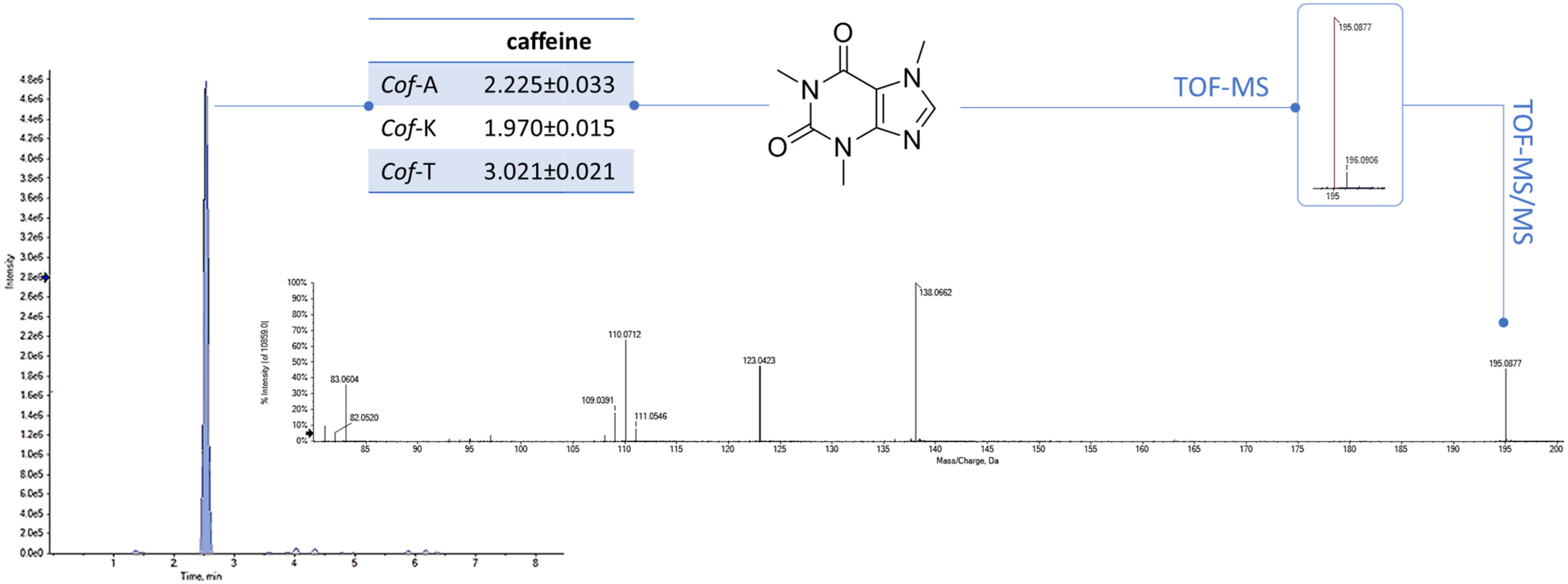

Figure S1. XIC, HR-MS and MS/MS of caffeine. In the table its amount is reported as wt % of dried moka coffee drinks (*Cof*-A = Coffee enriched with *C. sativa cv*. Antal shredded inflorescences; *Cof*-K = Coffee enriched with *C. sativa cv*. Kompolti shredded inflorescences; *Cof*-T = Coffee enriched with *C. sativa cv*. Tiborszallasi shredded inflorescences)

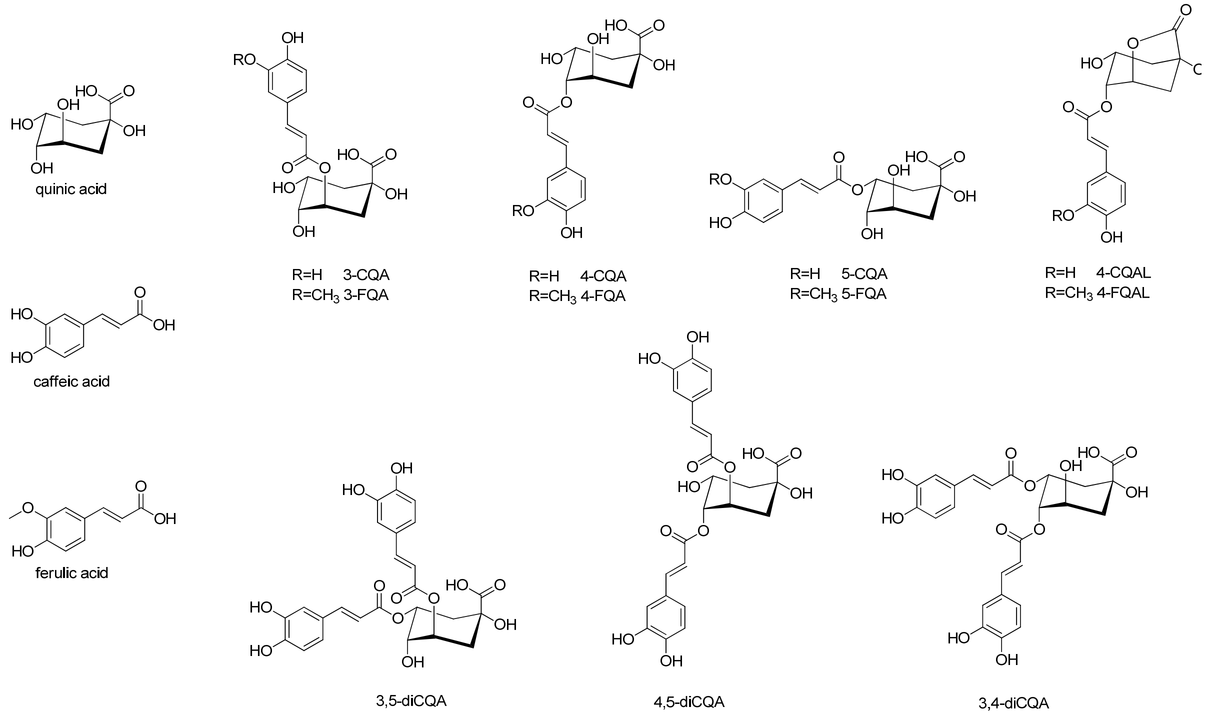

Figure S2. Chemical structures of the main chlorogenic acids (CQA = caffeoylquinic acid; diCQA = dicaffeoylquinic acid; CQAL = caffeoylquinic acid lactone; FQA = feruloylquinic acid; FQAL = feruloylquinic acid lactone).

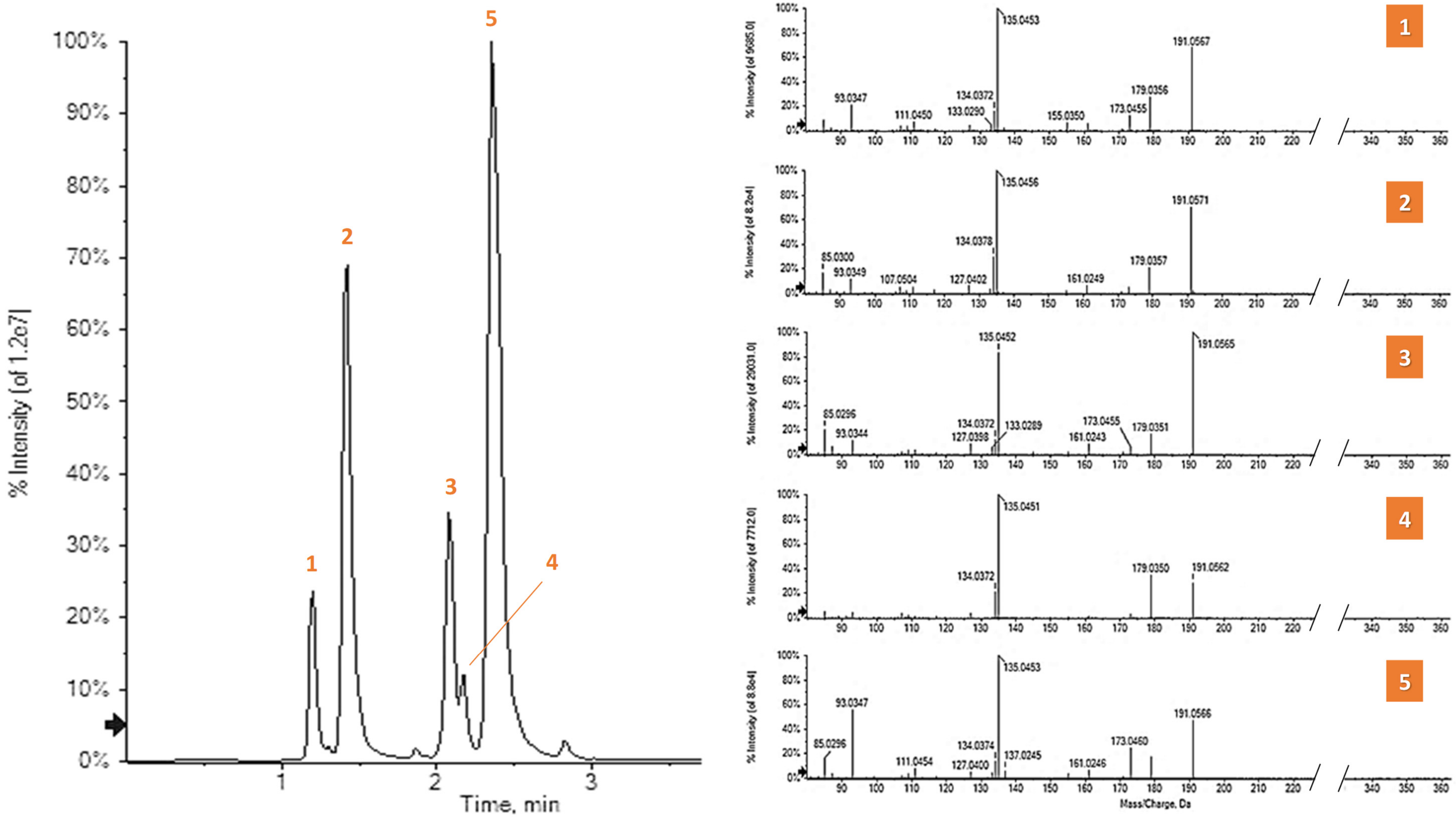

Figure S3. XIC (selecting the ion at *m/z* 353.09±0.025) and HR-MS/MS of monocaffeoylquinic acids (CQAs) (*Cof*-A = Coffee enriched with *C. sativa cv*. Antal shredded inflorescences; *Cof*-K = Coffee enriched with *C. sativa cv*. Kompolti shredded inflorescences; *Cof*-T = Coffee enriched with *C. sativa cv*. Tiborszallasi shredded inflorescences).

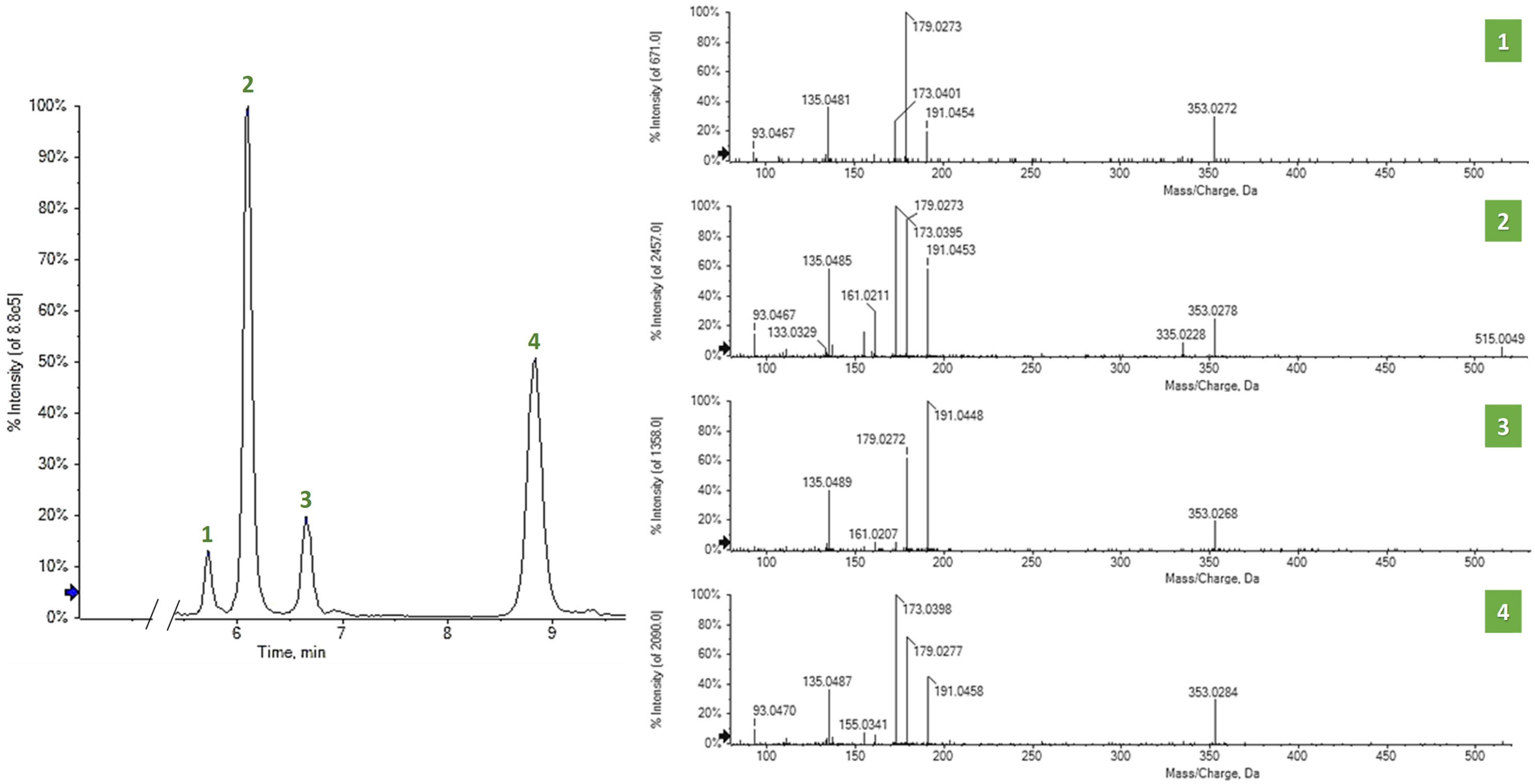

Figure S4. XIC (selecting the ion at *m/z* 515.10±0.025) and HR-MS/MS of di-caffeoylquinic acids (di-CQAs) (*Cof*-A = Coffee enriched with *C. sativa cv*. Antal shredded inflorescences; *Cof*-K = Coffee enriched with *C. sativa cv*. Kompolti shredded inflorescences; *Cof*-T = Coffee enriched with *C. sativa cv*. Tiborszallasi shredded inflorescences).

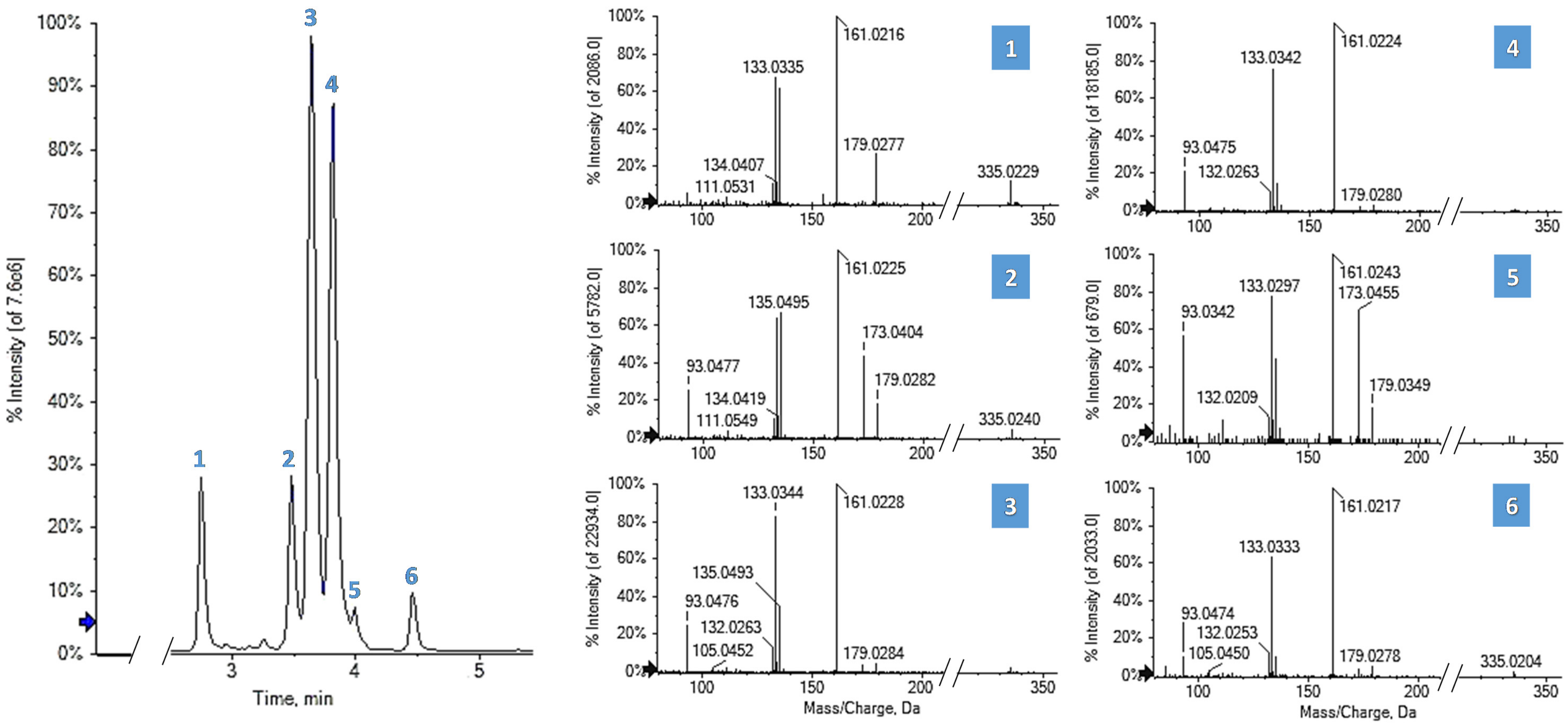

Figure S5. XIC (selecting the ion at *m*/*z* 335.08±0.025) and HR-MS/MS of dehydrated derivatives of caffeoylquinic acids (CSAs/CQALs) (*Cof*-A = Coffee enriched with *C. sativa cv*. Antal shredded inflorescences; *Cof*-K = Coffee enriched with *C. sativa cv*. Kompolti shredded inflorescences; *Cof*-T = Coffee enriched with *C. sativa cv*. Tiborszallasi shredded inflorescences).

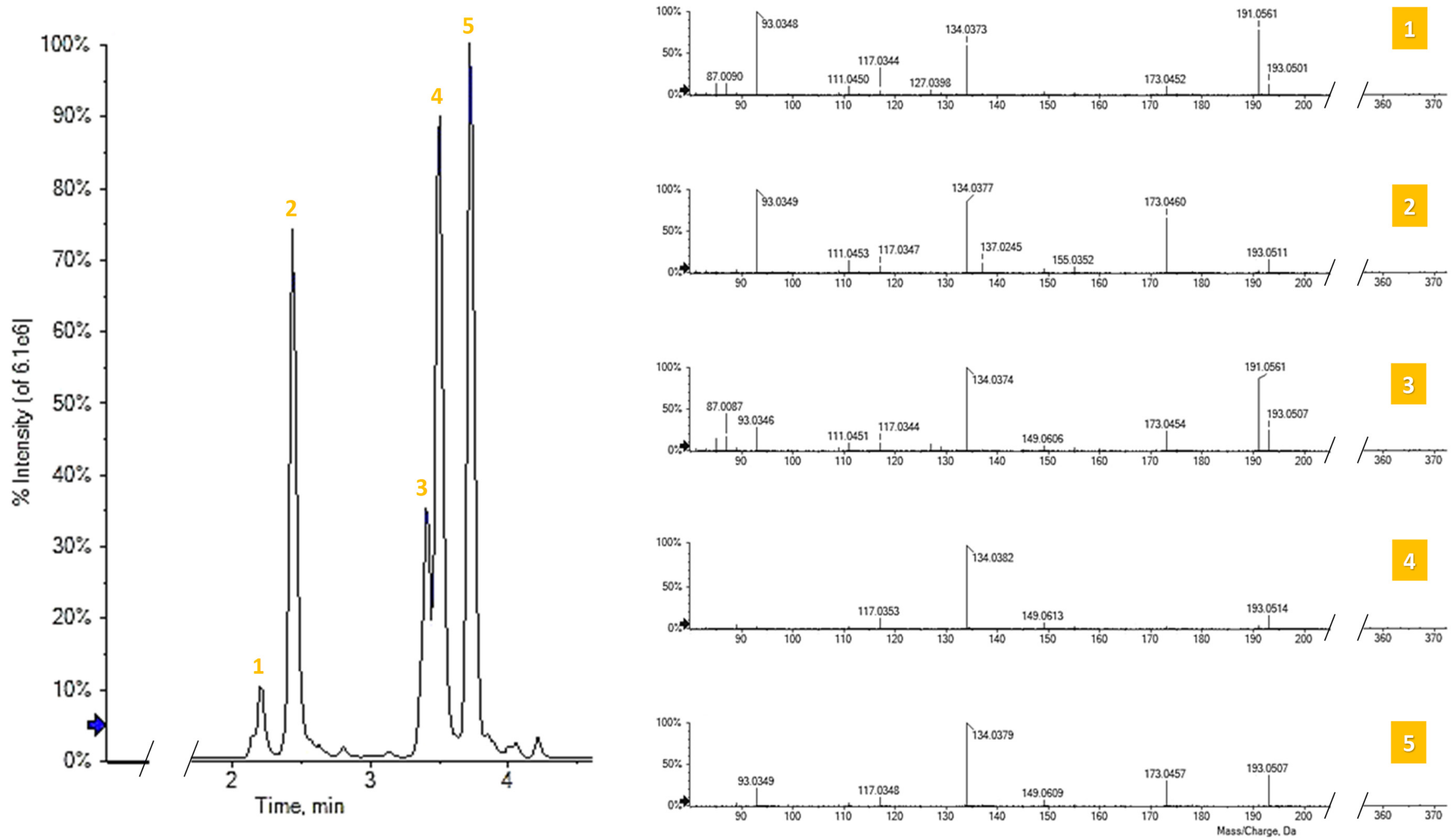

Figure S6. XIC (selecting the ion at *m*/*z* 367.08±0.025) and HR-MS/MS of feruloylquinic acids (FQAs) (*Cof*-A = Coffee enriched with *C. sativa cv*. Antal shredded inflorescences; *Cof*-K = Coffee enriched with *C. sativa cv*. Kompolti shredded inflorescences; *Cof*-T = Coffee enriched with *C. sativa cv*. Tiborszallasi shredded inflorescences).
